# Assessment of Urinary Ferritin as a Non-Invasive Diagnostic Test for Iron Deficiency Anemia in Pediatric Populations: A Case-Control Study

**DOI:** 10.30699/ijp.2025.2049356.3395

**Published:** 2025-11-12

**Authors:** Hossein Esfahani, Arya Derakhshesh, Ali Reza Soltanian, Hassan Bazmamoun, Alireza Rastgoo Haghi

**Affiliations:** 1 Department of Pediatric Hematology/Oncology, Hamadan University of Medical Science, Hamadan, Iran; 2 Student Research Committee, Hamadan University of Medical Sciences, Hamadan, Iran; 3 Department of Biostatistics, Hamadan University of Medical Sciences, Hamadan, Iran; 4 Department of Pediatric Gastroenterology, Hamadan University of Medical Sciences, Hamadan, Iran; 5 Department of Pathology, Hamadan University of Medical Science, Hamadan, Iran

**Keywords:** Iron-Deficiency Anemia, Serum Ferritin, Urine Ferritin

## Abstract

**Background & Objective:**

Iron deficiency anemia is the most prevalent form of anemia worldwide and can cause complications in children and adolescents. This study investigates the correlation between serum and urinary ferritin and evaluates the feasibility of using urinary ferritin to diagnose iron deficiency.

**Methods:**

In this case-control study, 45 patients with iron deficiency anemia were included in the case group and 45 healthy children in the control group. From each participant, 1.5 mL blood and 5 mL of urine were collected, and serum and urinary ferritin levels were measured using the chemiluminescent immunoassay (CLIA) method with Mindray kits. The results were analyzed and compared using SPSS software, version 16 (IBM Corp).

**Results:**

The mean age of patients was 7.95 years, and that of controls was 7.26 years. Female patients constituted 62.2% of the case group, and female controls represented 46.7% of the control group. In patients, the mean serum ferritin level was 13.39 ng/mL (SD = 6.37), and the mean urinary ferritin level was 3.50 ng/mL (SD = 2.65). In controls, the mean serum ferritin level was 63.7 ng/mL (SD = 41.8), and the mean urinary ferritin level was 3.98 ng/mL (SD = 2.89). Urinary ferritin demonstrated lower diagnostic accuracy for iron deficiency anemia compared with serum ferritin. The Spearman correlation coefficient between serum and urinary ferritin was 0.155, indicating a weak positive correlation.

**Conclusion:**

The findings of this study demonstrate an insignificant relationship between urine and serum ferritin levels. These findings indicate that urinary ferritin is not a reliable non-invasive alternative for diagnosing iron deficiency.

## Introduction

Iron is an essential micronutrient required for optimal brain function and overall health. Iron deficiency anemia (IDA) is the most prevalent form of anemia worldwide, affecting approximately one-third of the global population and occurring across all age groups (1). IDA is particularly common among children and adolescents, often resulting in long-term behavioral and neurological complications. Iron deficiency during infancy may cause persistent neurological and behavioral disturbances (2,3). Therefore, it is essential to monitor and identify iron deficiency in children exhibiting signs such as pallor, irritability, premature fatigue, muscular weakness, tachycardia, tachypnea, decreased attention, and organomegaly (4–6).

The Perl Prussian blue method is traditionally considered the gold standard for assessing iron levels in bone marrow; however, its invasive nature makes it unsuitable for frequent use, particularly in pediatric populations (7). Consequently, alternative biochemical markers, including serum ferritin (SF), serum iron concentration (SIC), total iron-binding capacity (TIBC), and transferring saturation (TS), have been used (8). Each of these markers, however, has limitations that may hinder accurate diagnosis (9). Ferritin, a cytoplasmic protein complex composed of iron and apoferritin, functions primarily in iron storage (10–15). Measurement of serum ferritin remains the standard laboratory method for detecting iron deficiency and requires venipuncture for blood collection (16–19).

However, obtaining blood samples from children can be challenging and may cause complications such as phlebitis (20). Therefore, a noninvasive and reliable laboratory test would be highly valuable. Several studies have investigated urinary ferritin measurement as a potential alternative (20–23); nevertheless, further research is required to validate its diagnostic accuracy.

This case-control study evaluates the validity of urinary ferritin measurement as a noninvasive approach for assessing iron deficiency in children with IDA, compared with serum ferritin levels. The study focuses on children aged 1 to 15 years who visited Besat Hospital in Hamadan between 2022 and 2023.

## Materials and Methods

This case-control study was conducted between 2022 and 2023 at Hamadan University of Medical Sciences and included 45 children diagnosed with iron deficiency anemia (IDA) and 45 healthy controls. Participants in the 2 groups were matched by age and sex.

The inclusion criteria for the case group were children aged 1 to 15 years with a confirmed diagnosis of IDA, defined by serum ferritin levels below 25 ng/mL and hemoglobin levels consistent with age- and sex-specific reference values. Participants were recruited from the pediatric hematology clinic of Besat Hospital and the Imam Khomeini Clinic. The control group included healthy children and adolescents aged 1 to 15 years without IDA and with normal serum ferritin levels.

Exclusion criteria for both groups included lack of parental consent, recent use of iron supplements, and the presence of acute or overt inflammatory symptoms, such as fever above 38 °C, urinary tract infection, or respiratory illness, based on the physician’s clinical assessment. Children who were generally unwell were also excluded.

For each participant, 1.5 mL of venous blood was drawn into ethylenediaminetetraacetic acid (EDTA) tubes for serum ferritin analysis, and 5 mL of midstream urine was collected in sterile containers for urinary ferritin determination. Serum and urine ferritin concentrations were measured using Mindray kits with the chemiluminescent immunoassay (CLIA) method. All samples were processed in the clinical laboratory of Hamadan University of Medical Sciences under standardized conditions to ensure analytical accuracy and reliability.

Data analysis was performed using SPSS software, version 16 (IBM Corp). Descriptive statistics were used to summarize demographic characteristics and ferritin levels. Spearman correlation analysis was applied to assess the relationship between serum and urinary ferritin levels. A *P* value <.05 was considered statistically significant.

The study was approved by the Ethics Committee of Hamadan University of Medical Sciences (approval code: IR.UMSHA.REC.1402.758). Written informed consent was obtained from the parents or legal guardians of all participants before sample collection.

## Results

In this study, 45 patients with iron deficiency anemia (IDA) and 45 healthy participants (control group) were included. The mean age of the case group was 7.95 years, and that of the control group was 7.26 years. In the case group, 62.2% were female and 37.8% were male, whereas in the control group, 46.7% were female and 53.3% were male.

The mean serum ferritin level in the IDA group was 13.39 ng/mL (SD = 6.37), which was significantly lower than that of the control group (63.7 ng/mL; SD = 41.8). The mean urinary ferritin level in the IDA group was 3.50 ng/mL (SD = 2.65), which was slightly lower than that of the control group (3.98 ng/mL; SD = 2.89) (Table 1).

**Fig. 1:**
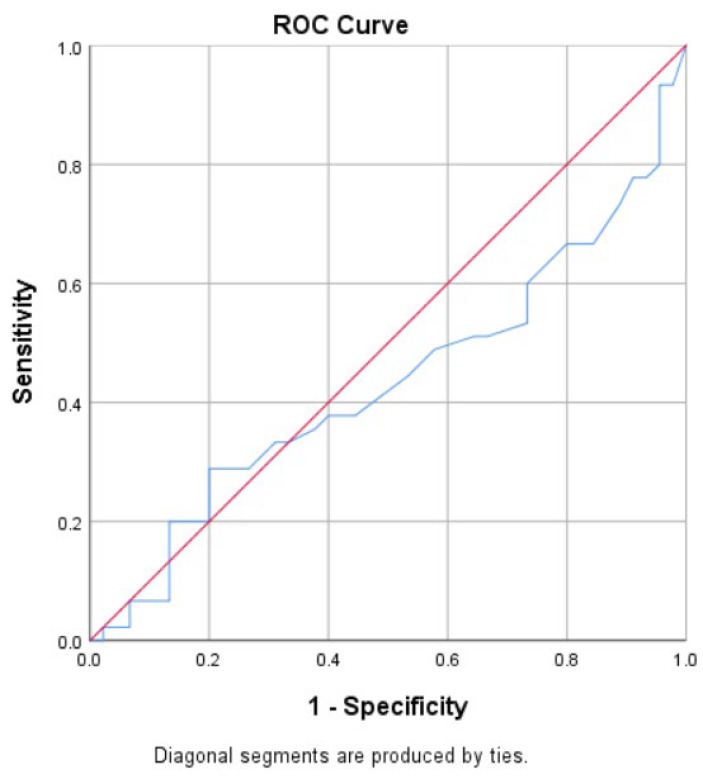
ROC Curve for Sensitivity and Specificity of Urine Ferritin Test

The optimal cutoff value for urinary ferritin was determined using receiver operating characteristic (ROC) curve analysis (Figure 1). The best cutoff value was 3 ng/mL. Based on Table 2, the sensitivity of urinary ferritin was 51%, specificity was 57%, the positive predictive value (PPV) was 53%, and the negative predictive value (NPV) was 54%.

**Table 1:** Laboratory Indexes and Age Characteristics in Case and Control Groups

Index	Case Group (n=45)	Control Group (n=45)
Serum Ferritin (ng/mL)	13.39 ± 6.37 (1.42 - 26.1)	63.7 ± 41.84 (26.06 - 201.5)
Urine Ferritin (ng/mL)	3.5 ± 2.65 (0.5 - 14.4)	3.98 ± 2.89 (0.5 - 15.6)
Age (years)	7.95 ± 5.48 (1 - 15)	7.26 ± 5.07 (1 - 15)
WBC (×1000/µL)	7.68 ± 1.89 (4.6 - 11)	7.52 ± 1.84 (4.06 - 11)
Hb (g/dL)	10.47 ± 1.21 (5.9 - 12.3)	13.24 ± 1.07 (11.2 - 16)
MCV (fl)	74.04 ± 8.44 (52.8 - 86)	83.31 ± 4.48 (75 - 93.2)
MCH (pg)	23.83 ± 3.6 (15.2 - 30.2)	28.48 ± 1.22 (26.2 - 32)
RBC (mil/µL)	4.98 ± 0.53 (3.87 - 6.32)	4.85 ± 0.57 (3.78 - 6.32)
PLT (×1000/µL)	332.87 ± 86.18 (112 - 694)	286.02 ± 73.29 (167 - 430)
RDW (% )	15.00 ± 1.22 (12.6 - 18)	12.92 ± 0.85 (11.2 - 14.7)

Values are presented as Mean ± SD (Min–Max).

**Table 2:** data’s for calculating Sensitivity, Specificity, Positive and Negative Predictive values for Urine Ferritin

Urine Ferritin	Disease Present	Disease Absent	Total
Test Positive	23	19	42
Test Negative	22	26	48
Total	45	45	90


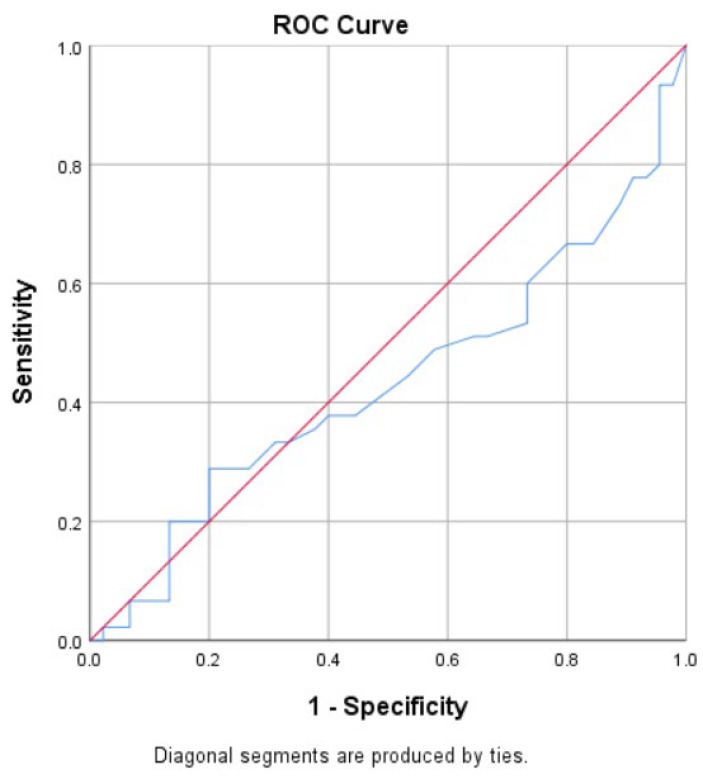


Spearman's correlation coefficient was used to assess the link between serum and urine ferritin levels. The study showed a slight positive connection (Spearman's coefficient = 0.155, p-value=0.145) but this correlation was not statistically significant. 

To evaluate whether urine ferritin levels differ significantly between individuals with and without iron deficiency, an independent-samples t-test was conducted. Levene’s test for equality of variances indicated that the assumption of equal variances was met (F = 0.076, p = 0.783), so the t-test results assuming equal variances were used. The analysis revealed no statistically significant difference in urine ferritin levels between the iron-deficient group (n = 45) and the control group (n = 45), with a t-value of -0.754 and a p-value of 0.453. The mean difference in urine ferritin between the two groups was -0.45 ng/mL (95% CI: -1.62 to 0.73), indicating that urine ferritin does not significantly differ by iron status and may not serve as a reliable standalone diagnostic marker. 

A Chi-square test was performed to evaluate the association between urine ferritin levels (categorized as low ≤3 ng/mL vs. normal >3 ng/mL) and iron deficiency status. The results showed no statistically significant association between low urine ferritin and iron deficiency [χ²(1) = 0.54, p = 0.462], indicating that low urine ferritin was not significantly more frequent in the iron-deficient group compared to controls. 

## Discussion

This study evaluated the diagnostic utility of urinary ferritin compared with serum ferritin in detecting iron deficiency anemia (IDA) among pediatric patients attending the Pediatric Hematology Clinic at Hamadan University of Medical Sciences from 2022 to 2023. The findings demonstrated that urine sampling in children is feasible and that ferritin was detectable in all urine samples using the employed chemiluminescent immunoassay (CLIA) method. However, the weak and statistically nonsignificant correlation between serum and urinary ferritin suggests that urinary ferritin alone is not a reliable indicator of iron status in children.

Furthermore, the results indicated that IDA can occur at any age and in both sexes within the studied population. Although the correlation between serum and urinary ferritin was weak and not statistically significant, this finding suggests that the observed association may be due to chance rather than a true physiological relationship. Larger studies are required to determine whether a genuine correlation exists. Additionally, the calculated sensitivity and specificity of urinary ferritin were low, supporting the conclusion that urinary ferritin is not an adequate substitute for serum ferritin in the diagnosis of pediatric IDA.

In a study by Gerday et al (23), a prospective cross-sectional design using a chemiluminescent immunoassay similar to that employed in the present study, the investigators examined premature infants with a gestational age of less than 37 weeks. In contrast, our study included children aged 1 to 15 years. The inclusion criteria of Gerday et al differed in that their cohort included participants with infections and inflammatory conditions, which may have affected the results because ferritin is an acute-phase reactant. In the present study, participants with evidence of infection or inflammation were excluded. Gerday et al corrected urinary ferritin levels for urinary creatinine, which improved sensitivity and specificity, and found that urinary ferritin levels below 10 to 12 ng/mL were statistically significant for diagnosing iron deficiency in infants. In contrast, no such correction was applied in the current study, which may explain the lack of diagnostic efficacy observed for urinary ferritin.

Similarly, Bahr et al (21) conducted a cross-sectional study of 24 participants, including healthy individuals, full-term newborns, preterm infants, and infants with iron overload disorders. However, their study did not use a standardized sampling protocol and included participants with chronic conditions such as β-thalassemia. The sample size was also considerably smaller than that of the present study. While Bahr et al measured serum and urinary ferritin levels using the enzyme-linked immunosorbent assay (ELISA) method, the current study used a chemiluminescent immunoassay. Bahr et al reported a correlation between serum and urinary ferritin levels based on Spearman correlation analysis, which contrasts with the findings of the present study.

Bazmamoun et al (22) conducted a cross-sectional study in Hamadan involving 76 low–birth-weight infants, in a setting similar to that of the present study. The inclusion criteria in their study, like ours, required the absence of inflammatory or viral illness. Both studies used the chemiluminescent immunoassay technique to measure serum and urinary ferritin levels and applied the Spearman correlation test to assess the relationship between the 2 variables. However, unlike our results, Bazmamoun et al reported a significant correlation between serum and urinary ferritin levels. Their study focused exclusively on infants younger than 28 days, whereas the present study included children aged 1 to 15 years. Among both full-term and preterm newborns, they found that serum and urinary ferritin levels were lower in premature infants but higher in male infants.

Ishikawa et al (24) reported a strong correlation between serum and urinary ferritin levels in a study of healthy individuals in Japan. Serum and urinary ferritin concentrations were significantly higher in men than in women. That study used a sandwich-type enzyme immunoassay (EIA) method to measure serum and urinary ferritin, which is a subset of the enzyme-linked immunosorbent assay (ELISA) and differs from the chemiluminescent immunoassay used in the present study. Based on the results of Ishikawa et al, urinary ferritin levels accounted for approximately 5% of serum ferritin concentrations.

Dewan et al (25), in a case-control study similar to the present one, investigated the efficacy of serum and urinary hepcidin in identifying iron deficiency anemia. The study included 30 children with iron deficiency anemia aged 6 months to 5 years in the case group and 30 nonanemic children in the control group, following a design similar to that of the current study. Dewan et al found no significant correlation between serum and urinary hepcidin levels; however, urinary hepcidin, unlike serum hepcidin, demonstrated acceptable specificity and sensitivity for detecting iron deficiency.

To explain the discrepancies between the findings of the present study and earlier research, it is important to consider the challenges associated with diagnosing iron deficiency, particularly when using newer approaches such as urinary ferritin testing. The limitations and difficulties encountered in the current study can help clarify these differences.

First, the systematic conditions of sample storage and collection for accurate ferritin measurement, especially in urine, may influence laboratory results. For example, in the study by Wolff et al (26), random urine samples were stabilized with bovine serum albumin (BSA) to improve accuracy, and the collection process was carefully controlled. In the studies by Gerday et al and Bazmamoun et al, sample collection was hospital based and standardized, whereas in the present study, urine samples, particularly from younger children, were collected on an outpatient basis, which may have affected results due to limited supervision.

Second, glomerular filtration rate (GFR), tubular reabsorption, and tubular secretion can influence urinary concentration (27). Adjusting urinary ferritin levels for urinary creatinine or specific gravity may yield more accurate results and strengthen the correlation with serum ferritin.

Third, ferritin is an acute-phase reactant whose levels may increase during infection or inflammation, potentially affecting results. In a study by Migliari et al (28), urinary ferritin levels were elevated in patients with bladder cancer. Although the current study excluded samples from participants with infection or inflammation based on medical history and the absence of leukocytosis in complete blood count (CBC) testing, additional laboratory markers—such as erythrocyte sedimentation rate (ESR) and C-reactive protein (CRP)—could improve the accuracy of sample selection. Nonetheless, the dependence of ferritin on inflammatory status limits its reliability for diagnosing and monitoring iron deficiency anemia.

Fourth, ferritin is composed of 24 subunits divided into 2 types: light (L) and heavy (H) chain proteins. It remains unclear whether urinary ferritin is excreted as an intact molecule or as fragments. Clarifying this mechanism may provide deeper insight into its diagnostic value.

Fifth, the standard laboratory instruments and commercial kits available for measuring urinary ferritin have not been adequately validated. Although urinary ferritin was detectable in all samples in the present study, its accuracy remains uncertain.

Finally, although the current study included a larger sample size and a broader age range than previous research, the sample population was still limited. The racial homogeneity of participants, mostly residents of Hamadan and neighboring regions, restricts the generalizability of the findings. Expanding the demographic and geographic diversity of future samples could enhance the robustness of the results.

To date, this is the only case-control study examining the relationship between urinary and serum ferritin levels specifically in children. The present investigation focused exclusively on evaluating urinary ferritin rather than performing a comprehensive iron panel. Serum ferritin was selected as the reference standard due to its well-established clinical utility and strong diagnostic value in assessing iron stores.

The clinical implications of this study suggest that, without further investigation, urinary ferritin cannot be considered a reliable biomarker for monitoring iron deficiency. Continued research into noninvasive diagnostic methods is essential. The present findings highlight the need for additional studies to better understand the underlying causes of urinary ferritin’s low correlation with serum ferritin and its limited diagnostic performance. Future investigations should explore ferritin metabolism and excretion, the influence of renal function on urinary ferritin levels, and the potential for combining urinary ferritin with other biomarkers to improve diagnostic accuracy.

## Conclusion

In conclusion, while our data did not support the basic idea that urine ferritin might be used as a non-invasive diagnostic tool for iron deficiency anemia (IDA) in children, this study does improve our understanding of the complexities involved in diagnosing IDA. The findings open the way for future study on refining diagnostic methodologies and developing novel non-invasive testing methods in pediatric populations.

## Data Availability

All data generated during the study are included in this article. Further enquiries can be directed to the corresponding author.
